# *Heterogeneous Nuclear Ribonucleoprotein L* is required for the survival and functional integrity of murine hematopoietic stem cells

**DOI:** 10.1038/srep27379

**Published:** 2016-06-07

**Authors:** Marie-Claude Gaudreau, Damien Grapton, Anne Helness, Charles Vadnais, Jennifer Fraszczak, Peiman Shooshtarizadeh, Brian Wilhelm, François Robert, Florian Heyd, Tarik Möröy

**Affiliations:** 1 Institut de recherches cliniques de Montréal (IRCM) 110 Avenue des Pins, Montréal, QC H2W 1R7, Canada; 2 Département de microbiologie, infectiologie et immunologie, Université de Montréal, C.P. 6128, succ. Centre-ville, Montréal, H3C 3J7, Canada; 3 Department of Experimental Medicine, McGill University, 1110 Pins Avenue West Room 101, Montréal, Quebec, H3A 1A3, Canada; 4 Institut de recherche en immunologie et cancerologie (IRIC), Université de Montréal, 2950 Chemin de Polytechnique, Marcelle-Coutu Pavilion, Montréal, Quebec, H3T 1J4, Canada; 5 Département de médecine, Faculté de médecine, Université de Montréal, Montréal, Canada; 6 Institut für Chemie und Biochemie, Freie Universität Berlin, Berlin, Germany

## Abstract

The proliferation and survival of hematopoietic stem cells (HSCs) has to be strictly coordinated to ensure the timely production of all blood cells. Here we report that the splice factor and RNA binding protein hnRNP L (heterogeneous nuclear ribonucleoprotein L) is required for hematopoiesis, since its genetic ablation in mice reduces almost all blood cell lineages and causes premature death of the animals. In agreement with this, we observed that hnRNP L deficient HSCs lack both the ability to self-renew and foster hematopoietic differentiation in transplanted hosts. They also display mitochondrial dysfunction, elevated levels of γH2AX, are Annexin V positive and incorporate propidium iodide indicating that they undergo cell death. Lin^-^c-Kit^+^ fetal liver cells from hnRNP L deficient mice show high p53 protein levels and up-regulation of p53 target genes. In addition, cells lacking hnRNP L up-regulated the expression of the death receptors *TrailR2* and *CD95/Fas* and show Caspase-3, Caspase-8 and Parp cleavage. Treatment with the pan-caspase inhibitor Z-VAD-fmk, but not the deletion of p53, restored cell survival in hnRNP L deficient cells. Our data suggest that hnRNP L is critical for the survival and functional integrity of HSCs by restricting the activation of caspase-dependent death receptor pathways.

In mice, hematopoiesis originates from hematopoietic stem cells (HSC) that migrate from the aorta-gonad-mesonephros region (AGM) towards the fetal liver (FL) at embryonal stage 10.5 day post-coitus and later on, takes place in the bone marrow (BM) of adult mice^1^,^2^. In both FL and BM, HSCs possess a unique self-renewal capacity and the potential to generate all mature blood and immune cells of an organism throughout its lifetime^3^,^4^,^5^. The commitment of HSCs to differentiate into specific cell lineages is tightly regulated and starts with the formation of multipotent progenitors (MPPs) that have a reduced self-renewal capacity and are already restricted in their multilineage potential^6^,^7^. The earliest precursors that emerge from MPPs still have both myeloid and lymphoid potential and are called LMPPs^8^,^9^.

HSCs reside in the BM or the FL and are part of the Lin^−^Sca1^+^cKit^+^ (LSK) subset. They can be further defined by the expression of the markers CD150 and CD48 (i.e. HSCs are Lin^−^Sca1^+^cKit^+^CD150^+^CD48^−^)^10^,^11^,^12^,^13^. While most HSCs in adult mice are in a quiescent stage, embryonic HSCs are proliferating to generate the adult pool of stem cells^5^,^14^,^15^. Many transcription factors including Runx1, Gfi1, Gfi1b, GATA2, SCL and Notch1 have been identified as important regulators of lineage commitment as well as HSCs quiescence and survival^16^,^17^,^18^,^19^,^20^. However, the role that mRNA processing factors may have for HSCs remains unexplored, even though they are known to control gene expression at the transcriptional and posttranscriptional level^21^,^22^.

Heterogeneous nuclear ribonucleoprotein L (hnRNP L) is an RNA processing factor and an RNA-binding protein that has been identified to regulate alternative splicing by binding exonic splicing silencers elements (ESS) resulting in exon exclusion from the mature mRNA^23^,^24^,^25^. To investigate the role of hnRNP L in HSC function and hematopoietic differentiation, we have generated conditional hnRNP L knockout mice. Here, we present evidence that hnRNP L is essential for the survival and functional integrity of HSCs since ablation of this factor is incompatible with proper hematopoietic differentiation and causes premature and accelerated death in hnRNP L deficient animals. In particular, we report that hnRNP L deficient HSCs show increased mitochondrial stress and initiate both p53- and caspase-dependent cell death pathways.

## Material and Methods

### Ethics Statement

The protocols for the *in vivo* experiments described here were reviewed and approved by the Institut de recherches cliniques de Montréal (IRCM) Animal Care Committee (ACC); protocol numbers are: #2009-12/#2013-03. All animal experiments were conducted according to institutional rules put in place by the IRCM ACC, which follow the regulations and requirements of the Canadian Council on Animal Care (www.ccac.ca).

### Mice

hnRNP L floxed mice were described previously^26^. *MxCre, VavCre, H2kBcl2* and *Trp53*^−/−^ mice were obtained from Jackson laboratories or from a colony maintained at the IRCM. The animal ethics committee of the IRCM approved all animal experiments. MxCre^+^hnRNP L^fl/fl^ or hnRNP L^fl/fl^ mice were injected intraperitoneally with 500 μg of polyinosinic-polycytidylic acid (pIpC; Sigma-Aldrich) every other day for a total of 5 times and were analyzed 14 days after the first injection. All mice were aged between 8–16 weeks for BM analysis.

### Flow cytometric analysis and cell sorting

Cell surface staining was done using a lineage cocktail containing antibodies against B220, Ter119, CD3, CD11b, Gr1, NK1.1, CD49b and CD8 that were all biotinylated and streptavidin labeled with a fluorescent dye (BD biosciences). Importantly, no antibody against CD11b was included in the lineage cocktail for FL cells. Antibodies against CD150, CD48, cKit, Sca1, CD34, Flt3, CD16/32, AA4.1, IL7R, Annexin V, CD95 and TrailR2 were either from BD biosciences, ebiosciences or Biolegends. Biotinylated Sca-1 antibody was included in the lineage cocktail mix when staining for myeloid progenitor cells. Hoechst staining and the analysis of reactive oxygen species (ROS) was performed as previously described^17^. Briefly, cells were first surface stained with specific markers and then incubated at 37 °C for 3 min with 5-(and-6)-carboxy-2′,7′-dichlorodihydrofluorescein diacetate (carboxy-H2DCFDA; Invitrogen) and analyzed by fluorescence activated cell sorting. Staining for phosphorylated H2AX was performed by fixing the cells with 2% paraformaldehyde and by permealizing with PhosphoFlow buffer III (BD biosciences) for 20 min on ice. Cells were then stained with anti-γH2AX (Cell signaling) followed by a FITC coupled anti-rabbit antibody. All analyses were done using a LSR FACS (BD biosciences). Mitochondria membrane potential was measured by fluorescence levels upon staining with Mitotracker green and Mitotracker deep red at 50 nM for 30 min at 37 °C, using an Invitrogen kit and performed according to the manufacturer’s instructions. LSK cells were sorted on a MoFlo cell sorter (Cytomation). Peripheral cells were stained with fluorescent conjugated antibodies against CD3, B220, CD19, Mac1, Gr1, Ter119, CD71 that were all from BD biosciences.

### *In vitro* differentiation

OP9 or OP9DL1 cells were plated in AMEM with either IL-7 and SCF or IL-7, SCF, GM-CSF, IL-3 and IL-6 at a density of 2 × 10^4^ cells in 24-well plates. Two thousand LSK cells from FL of E14.5 embryos were sorted into each well. Cells were harvested 7 or 14 days later and were stained for CD4, CD8, CD19, Gr1 and Mac1.

### Methylcellulose assay

Five hundred LSK cells sorted from E14.5 FL or BM were seeded on methycellulose (StemCell Technologies) supplemented with erythropoetin, IL-3, IL-6, SCF, transferrin and insulin. After 10 days, the number of colonies was determined.

### Treatment with inhibitors

5 × 10^4^ Lin-FL cells from embryos E13.5 were sorted using an AutoMACS into StemSpan (StemCell Technologies) culture media supplemented with 2.6% FBS, L-Glutamine and SCF. The caspase-8 (Z-IETD-fmk) and Pan-caspase (Z-VAD-fmk) inhibitors were purchased from R&D Systems and used at a final concentration of 100 μM. The ATM (KU-55933) and ATR (VE-822) inhibitors were purchased from Selleck Chemicals and used at a final concentration of 1nM. Cells were cultured for 24 hours before apoptosis rates were measured by annexin V staining (Annexin V-FITC Apoptosis Detection Kit; BD Pharmingen).

### Transplantation assays

Non-competitive repopulation assays were performed using total FL cells from embryos E14.5 (2 × 10^5^) or total BM (1 × 10^6^). Competitive transplantation assays were done by pooling total BM cells from MxCre^+^hnRNPL L^fl/fl^ or MxCre^+^hnRNP L^wt/fl^ (CD45.2^+^) or FL cells from VavCre^+^hnRNP L^fl/fl^ or hnRNP L^wt/fl^ (CD45.2^+^) with BM cells from wt CD45.1^+^ mice at a 1:1 ratio for a total of 2 × 10^6^ cells and 4 × 10^5^, respectively. FL Lin^−^cKit^+^ progenitor cells transplant experiment was done by pooling 7,500 VavCre^+^hnRNP L^fl/fl^ or hnRNP L^wt/fl^ (CD45.2^+^) FL Lin^−^cKit^+^ progenitor cells with 1.5 × 10^5^ carrier CD45.1^+^ FL cells. Cells from donor mice aged 8–12 weeks (one donor mouse per experiment) were injected intravenously into 9.6 Gy irradiated syngeneic 8–12 week old CD45.1^+^ mice.

### Real-time quantitative PCR

Total RNA was isolated from lineage depleted FL or BM cells using RNeasy Mini kit (Qiagen) according to the manufacturer’s instructions. cDNA was prepared from RNA using SuperScript II reverse transcriptase (Invitrogen) and was analyzed using TaqMan probes (Applied biosystems) and Gapdh as an internal control. A Mx-3005 system (Stratagene) was used and relative expression was calculated via the 2^−ΔΔCT^ method. QPCR was performed using a MasterMix for SYBR green (Quanta Biosciences) and a ViiA7 (Applied Biosystem). Melting curves were used to confirm the amplicon specificity. The exon usage was calculated via the ΔΔCT (variable exon- constant exon) and the 2^−ΔΔCT^ method. Primer sequences are provided in Supplementary Table 1.

### RNA-Sequencing

RNA was extracted from sorted FL Lin^−^, c-Kit^+^ cells of E14.5 embryos using MagMAX™-96 Total RNA Isolation Kit. RNA integrity and quality were confirmed using a bioanalyzer (Agilent). Sequencing libraries were prepared from RNA extracts from three biological replicates using the TruSeq Stranded mRNA kit from Illumina according to the manufacturer’s instructions and sequenced using the TruSeq PE Clusterkit v3-cBot-HS on an Illumina HiSeq 2000 system. Sequencing reads were aligned to the GRCm38 Ensembl build of the genome using Tophat v2.0.10^27^. On average, each sample had 170350714 mapped reads with a standard deviation of 21743591. For differential expression, reads were processed using Samtools^28^ and mapped to Ensembl transcripts using HTSeq^29^. Differential expression was tested using the DESeq R package and heatmaps were generated using the gplots R package^30^. Raw RNA-sequencing results and processed data are available on the Gene Expression Omnibus under accession GSE57875. The DAVID online tool was used to identify over-represented biological functions among genes significantly differentially expressed between hnRNPL KO vs. Wild-type samples^31^,^32^. The enrichment of selected biological functions of interest was also analyzed using the GSEA tool^33^.

### Splicing Analysis

For the analysis of splice junctions, processed reads were mapped to exon junctions and differences between conditions were quantified using the MISO software^34^. For the analysis of Differential Exon Usage (DEU), reads were mapped to exons using Dexseq_count.py script and quantified using the DEXseq R package. These two analyses differ in that MISO focuses on sequencing reads spanning exon junctions while Differential Exon Usage focuses on relative differences in the abundance of reads within exons, thereby producing different results.

### ChIP-qPCR

The detection of hnRNP L by ChIP was performed, as described previously^35^, on 5 × 10^6^ NIH3T3 cells cross-linked with 1% formaldehyde for 8 minutes and quenched with 125 mM glycine. Cells were lysed and chromatin was sonicated (Covaris E220 sonicator) to a size range of 200–500 bp. Samples were immunoprecipitated with 5μg anti-hnRNP L antibody (Ab32680; Abcam) or control anti-IgG antibody (sc-2025, Santa Cruz Biotechnology) coupled with Dynabeads beads coated with Protein G (Life Technologies). ChIP DNA was analyzed by quantitative PCR (qPCR) using SYBR green. The enrichment (relative to Input and a negative control intergenic region) was calculated using the ΔΔCt method. Primer sequences are provided in Supplementary Table 1.

### Luciferase reporter assays

HEK293T cells were plated on 24-well plates at a density of 1 × 10^5^ cells per well and, 24 h and 48 h later, transfected with 2pmol of siRNA using Lipofectamine 2000 as per the manufacturer’s instructions. At the 72 h time point, cells were transfected with both 250 ng the *Trailr2*^36^ or control luciferase reporters and β-galactosidase, which was used to normalize luciferase values in each well. Firefly luciferase and beta-galactosidase activities were measured 24 h post-reporter transfection.

### Actinomycin D assay

FL Lin^−^ cells were sorted from E14.5 embryos into serum-free opti-MEM (Gibco). Cells were then treated with 5 μg/mL actinomycin D at 37 °C and collected at 0, 2 and 4 hours post-treatment. Cells were lysed in RLT buffer with 10% β-mercaptoethanol and RNA was extracted using the Qiagen RNEasy Mini Kit, followed by RT-PCR and qPCR.

### Statistical analysis

Two-tailed Student *t* test was used to calculate *p* values where indicated. A *p* value ≤0.05 was considered statistically significant: *p ≤ 0.05, **p ≤ 0.01, ***p ≤ 0.001. Statistical analysis of survival curves was performed using the Log-rank test.

## Results

### Absence of hnRNP L results in altered hematopoiesis and premature death

**Figure 1:**
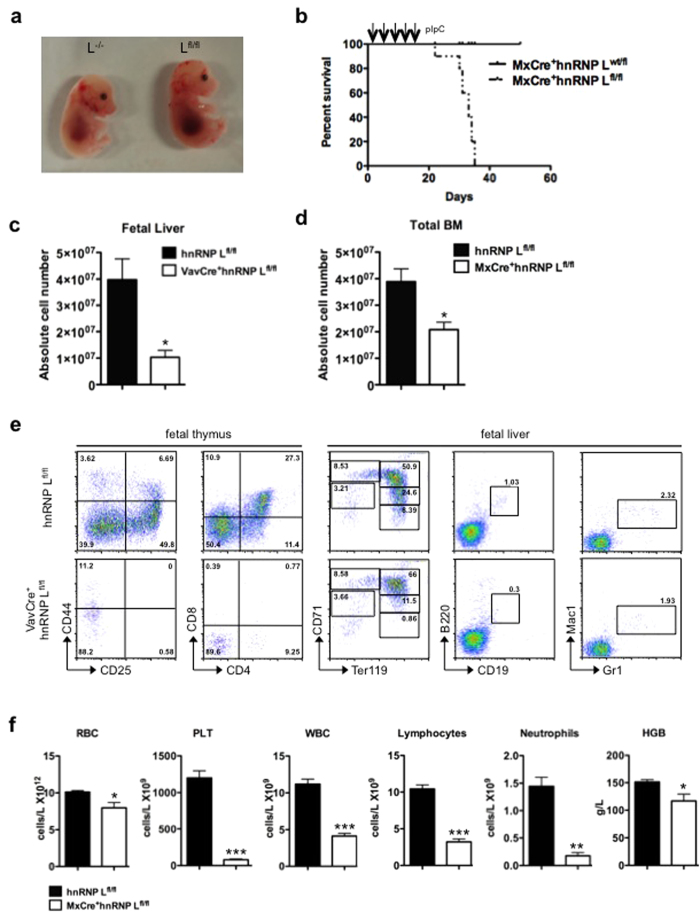
Impaired hematopoiesis in the absence of hnRNP L. (**a)** Representative image of control (L^fl/fl^) and VavCre^+^hnRNP L^fl/fl^ (L^−/−^) embryos at 14.5 days of embryonic development. (**b)** Survival curve of control and MxCre^+^hnRNP L^fl/fl^ mice injected five times with 500 μg of pIpC. (**c)** Histogram showing absolute cell numbers of control and VavCre^+^hnRNP L^fl/fl^ embryos at E14.5 in whole FL or (**d)** MxCre^+^hnRNP L^fl/fl^ in total BM from two tibia and two femur bones. (n = 6 or more). (**e)** Flow cytometric analysis of lymphoid, erythroid and myeloid subsets in fetal thymus or FL from hnRNP L^fl/fl^ or VavCre^+^hnRNP L^fl/fl^ deleted embryos at E14.5. FACS plots are representative of three independent experiments. (**f)** Analysis of blood parameters from adult control or MxCre^+^hnRNP L^fl/fl^ mice performed using an Advia system (RBC: red blood cell, PLT: platelets, HGB: hemoglobin, WBC: white blood cells). (n = 6 or more). All error bars are means ± SEM (*p < 0.01, **p < 0.001, ***p < 0.0001).

Since constitutive deletion of hnRNP L arrests development before gastrulation, we had previously generated conditional knockout mice carrying floxed hnRNP L alleles^26^. To gain insight into the function of hnRNP L in hematopoietic stem and progenitor cells (Figure S1a), we deleted the floxed alleles using a Vav-Cre transgene^37^. However, VavCre^+^hnRNP L^fl/fl^ mice were not viable and arrested development at embryonic stage E16.5-17.5 (Fig. 1a). To overcome this limitation, hnRNP L^fl/fl^ mice were crossed with animals carrying the pIpC inducible Mx-Cre transgene, but an acute ablation of hnRNP L by pIpC injection also caused MxCre^+^hnRNP L^fl/fl^ mice to die quickly (Fig. 1b). Both the fetal liver (FL) of VavCre^+^hnRNP L^fl/fl^ mice and the bone marrow (BM) of adult MxCre^+^hnRNP L^fl/fl^ mice had a significantly reduced cellularity (Fig. 1c,d) accompanied by a reduction of lymphoid and erythroid cells in the FL and an almost complete loss of T cells from the fetal thymus (Fig. 1e). Analysis of peripheral blood of adult pIpC injected MxCre^+^hnRNP L^fl/fl^ mice also showed a decrease of differentiated cells including severe thrombocytopenia and neutropenia (Fig. 1f). This diminution was observed although the ablation of hnRNP L in adult BM was less efficient than in the FL (Figure S1b). These findings indicate that ablation of hnRNP L causes a severe disruption of hematopoiesis at very early stages, possibly at the level of hematopoietic stem cells.

### Differential requirement of hnRNP L for fetal and adult HSCs and progenitors

**Figure 2:**
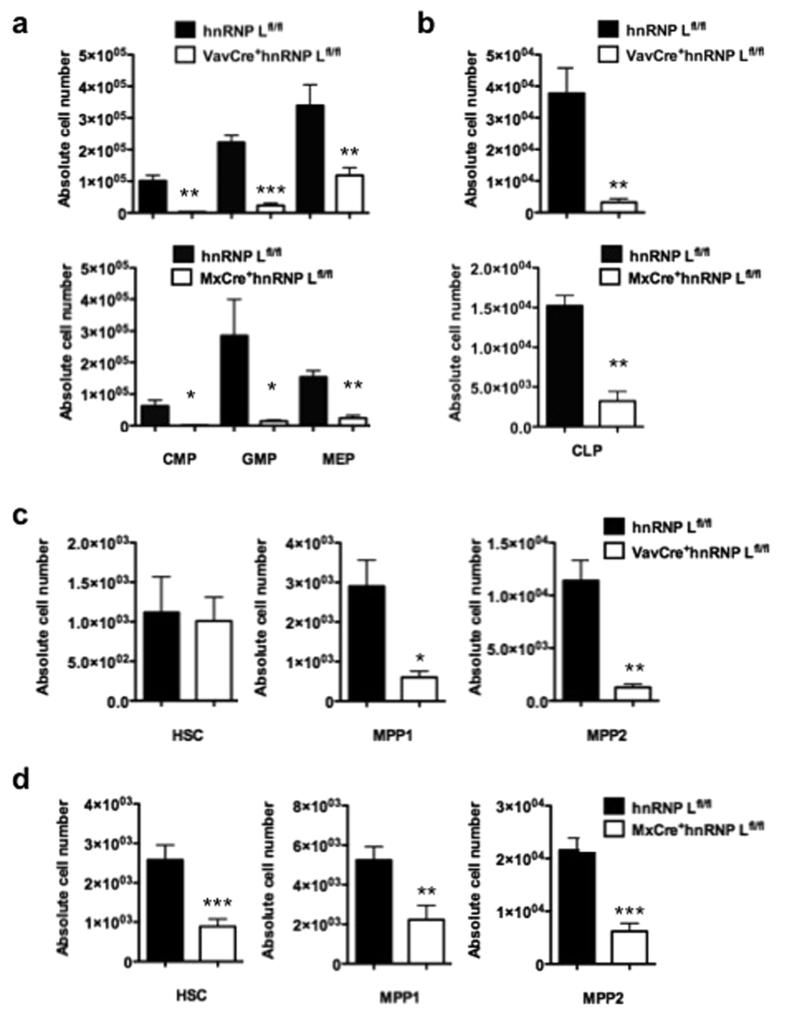
Impaired hematopoiesis and loss of progenitors and HSCs in FL and BM of hnRNP L deleted mice. (**a)** Absolute cell numbers of granulo-myeloid-erythroid progenitors GMP (defined as Lin^−^c-Kit^+^Sca-1^−^CD34^+^CD16/32^+^), CMP (defined as Lin^−^c-Kit^+^Sca-1^−^CD34^+^CD16/32^−^) and MEP (defined as Lin^−^c-Kit^+^Sca-1^−^CD34^−^CD16/32^−^). Shown are data from control mice (hnRNP L^fl/fl^) or animals where hnRNP L was deleted in FL (VavCre^+^hnRNP L^fl/fl^) or in adult BM (MxCre^+^hnRNP L^fl/fl^) (n = 3 or more). (**b)** Absolute cell numbers of common lymphoid progenitor (CLP) in both FL (n = 5) and BM (n = 6) of control or hnRNP L deficient mice. (**c)** Absolute cell numbers of HSCs (defined as Lin^−^Sca1^+^c-Kit^+^ (LSK) Flt3^−^CD150^+^CD48^−^), MPP1 (defined as LSK Flt3^−^CD150^+^CD48^+^) and MPP2 (defined as LSK Flt3^−^CD150^−^CD48^−^) from FL control mice (hnRNP L^fl/fl^) and VavCre^+^hnRNP L^fl/fl^. (**d)** Absolute cell numbers of HSCs, MPP1 and MPP2 from BM of control mice (hnRNP L^fl/fl^) and MxCre^+^hnRNP L^fl/fl^ (n = 3 or more). All error bars are means ± SEM (*p < 0.01, **p < 0.001, ***p < 0.0001).

Flow cytometric analysis revealed a significant reduction in frequencies and absolute numbers of common lymphoid and myeloid progenitors (CLPs, CMPs) and granulocytic monocytic progenitors (GMPs) and to a lesser extent of erythroid/megakaryocytic precursors (MEPs) in both FL and BM of hnRNP L deficient mice compared to controls (Fig. 2a,b, Figure S1c–f). Furthermore, the LSK fraction was severely affected by the deletion of the hnRNP L gene (Figure S1g), but absolute numbers of HSCs (LSK, Flt3^−^CD150^+^CD48^−^ cells) were maintained compared to controls (Fig. 2c). However, in the BM of adult MxCre^+^hnRNP L^fl/fl^ mice, frequencies and absolute numbers of both HSCs and the MPP progenitors were significantly reduced compared to controls (Fig. 2d, Figure S1h). This suggests that hnRNP L is required to maintain the cellularity of phenotypically defined hematopoietic stem and progenitor populations, but also indicates that hnRNP L ablation has different effects in cells derived from FL or BM.

### Phenotypically defined HSCs from hnRNP L deficient mice are functionally impaired

**Figure 3:**
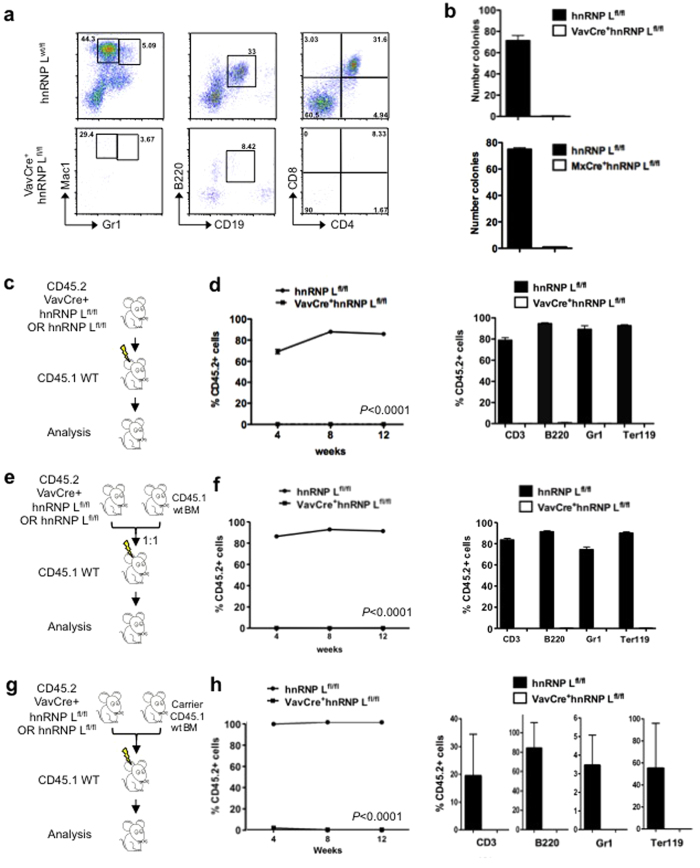
HSCs require hnRNP L to be functional. (**a)** Differentiation assays on OP9 and OP9-DL1 stromal cells; representative dot plots of myeloid cells (Gr1^+^Mac1^+^), B-cells (B220^+^CD19^+^) and T-cells (CD4^+^CD8^+^) differentiation of FL LSK cells from control (upper panel) and VavCre^+^hnRNP L^fl/fl^ mice (lower panel) (n = 3) (**b)** Colony assay on methylcellulose; 500 LSK cells from FL or BM from the indicated mice were seeded on methylcellulose and colonies were counted after 10 days of culture (n = 3). (**c)** Non-competitive transplantation assay: 2 × 10^5^ FL cells of either VavCre^+^hnRNP L^fl/fl^ or control (hnRNP L^fl/fl^) stage E14.5 embryos (both CD45.2^+^) were transplanted into irradiated syngenic CD45.1^+^ mice and the percentage of total CD45.2^+^ cells in the blood were measured at 4, 8 and 12 weeks post-transplantation. (**d)** The frequency of CD45.2^+^ cells within the T-, B-, myeloid or erythroid subpopulations (i.e. within CD3, B220, Gr1, Ter119 positive cells) in the blood of the recipient mice 12 weeks post-transplantation was assessed (n = 6). (**e)** Competitive transplantation assay: 2 × 10^5^ FL cells from VavCre^+^hnRNP L^fl/fl^ or VavCre^+^hnRNP L^wt/fl^ were mixed with 2 × 10^5^ normal CD45.1^+^ BM cells at a ratio of 1:1 for a total of 4 × 10^5^ cells. The mixtures were transplanted into irradiated CD45.1^+^ recipient mice and the percentages of CD45.2^+^ cells in the blood were measured at 4, 8 and 12 weeks post-transplantation. (**f)** The frequency of CD45.2^+^ cells within the T-, B-, myeloid or erythroid subpopulations (i.e. within CD3, B220, Gr1, Ter119 positive cells) in the blood of the recipient mice 12 weeks post-transplantation was assessed (n = 3). (**g)** 7.5 × 10^4^ Lin^−^cKit^+^ progenitor cells from VavCre^+^hnRNP L^fl/fl^ or VavCre^+^hnRNP L^wt/fl^ were mixed with 2 × 10^5^ normal carrier CD45.1^+^ BM cells. The mixtures were transplanted into irradiated CD45.1^+^ recipient mice and the percentages of CD45.2^+^ cells in the blood were measured at 4, 8 and 12 weeks post-transplantation. (**h)** The frequency of CD45.2^+^ cells within the T-, B-, myeloid or erythroid subpopulations (i.e. within CD3, B220, Gr1, Ter119 positive cells) in the blood of the recipient mice 12 weeks post-transplantation was assessed (n = 3). All error bars are means ± SEM.

**Figure 4:**
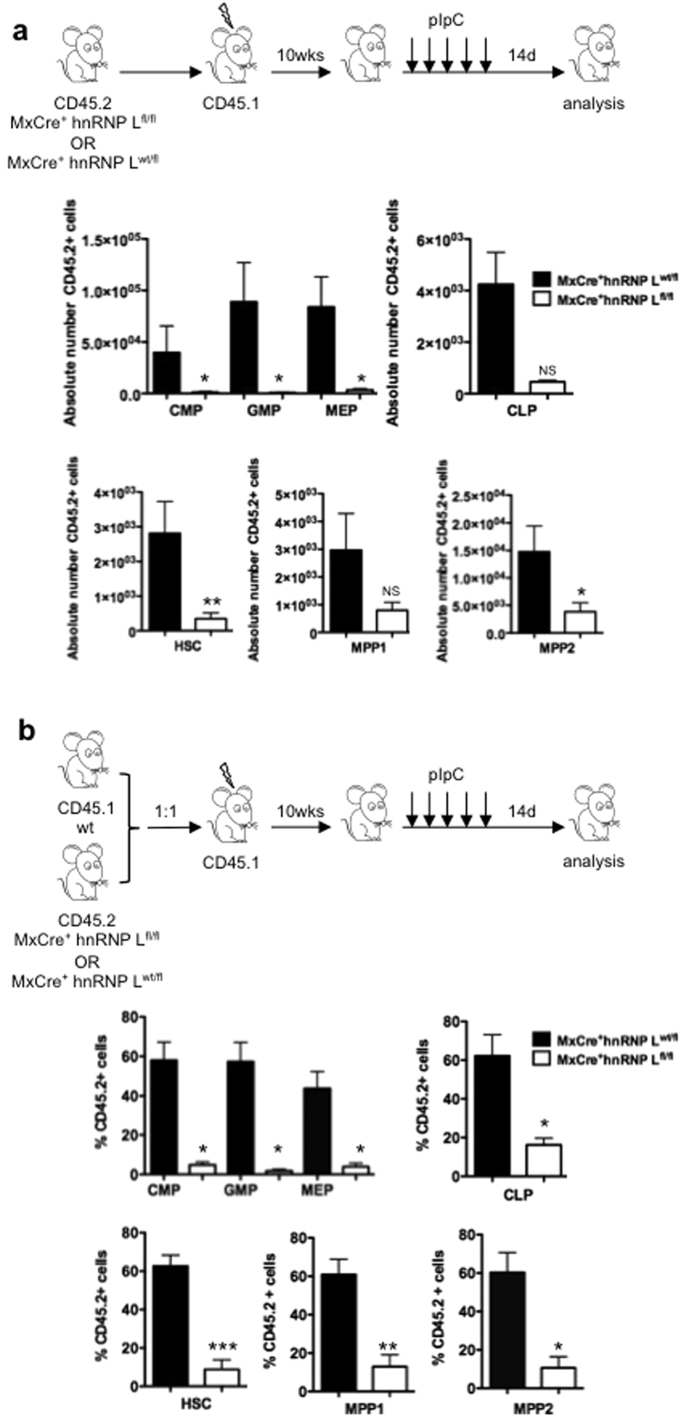
hnRNP L is required for HSCs and progenitor cells repopulation capacity. (**a)** Transplantation of total BM cells from MxCre^+^hnRNPL^wt/fl^ or MxCre^+^hnRNPL^fl/fl^ mice into irradiated syngenic CD45.1^+^ recipient mice. Ten weeks post-transplantation reconstitution was tested and mice were injected five times every other day with 500μg of pIpC and analyzed 14 days later. The frequency of CD45.2^+^ cells and absolute cells numbers were quantified for progenitors and HSCs in the BM (n = 4). (**b)** Competitive transplantation of total BM cells from MxCre^+^hnRNPL^wt/fl^ or MxCre^+^hnRNPL^fl/fl^ mice mixed with wt CD45.1^+^ BM cells at a ratio of 1:1 for a total of 2 × 10^6^ cells into irradiated syngenic CD45.1^+^ recipient mice and treated the same as the mice in A. (n = 4). All error bars are means ± SEM (*p < 0.01, **p < 0.001, ***p < 0.0001).

Using OP9 and OP9DL1 stroma co-cultures and methylcellulose assays, we found that hnRNP L deficient LSKs were unable to generate B- or T- lymphoid or myeloid cells in culture or any type of colony in semisolid medium, whereas wt LSKs efficiently gave rise to all three lineages and produced the expected number of colonies (Fig. 3a,b). Next, we transplanted non-competitively (Fig. 3c,d) or competitively (Fig. 3e,f) FL cells or transplanted FL Lin^−^cKit^+^ progenitor cells (Fig. 3g,h) from either wt or hnRNP L deficient embryos (CD45.2^+^) into irradiated syngeneic recipient mice (CD45.1^+^). Analysis of peripheral blood of the recipients showed that hnRNP L deficient cells were unable to generate detectable numbers of CD45.2^+^ cells upon transplantation, whereas transplanted control cells demonstrated a very efficient reconstitution of all lineages (Fig. 3d,f,h). Finally, we used cells from MxCre^+^hnRNP L^wt/fl^ or MxCre^+^hnRNP L^fl/fl^ mice and induced the deletion of hnRNP L in verified BM transplant chimeric mice after a competitive transplantation into syngeneic CD45.1^+^ mice (Figure S2a,b). Clearly, transplanted mice showed a strongly reduced percentage of CD45.2^+^ progenitors and HSCs after the deletion of hnRNP L alleles (Fig. 4a,b), suggesting that functional HSCs are almost entirely lost from hnRNP L deficient FL or BM.

### Loss of hnRNP L leads to HSC apoptosis and mitochondrial stress

**Figure 5:**
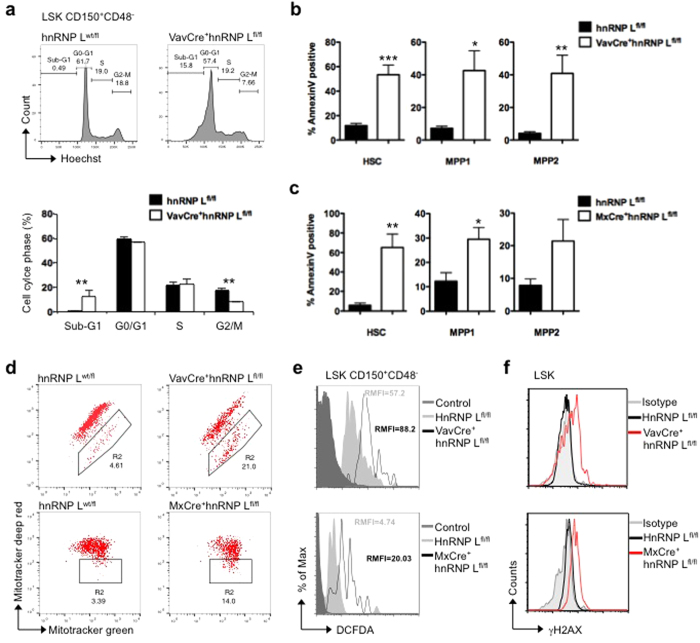
Apoptosis induced by hnRNP L deletion in HSCs and progenitor cells. (**a)** HSCs (defined as LSK Flt3^−^, CD150^+^CD48^−^) from FL of animals with the indicated genotype were analyzed by Hoechst staining (upper panel). The percentages of cells in the different cell cycle phases are indicated by the gates in the histograms and the bar graph (lower panel). (n = 3). (**b,c)** Cell death was assessed by Annexin V staining in HSCs (defined as LSK Flt3^−^, CD150^+^CD48^−^), MPP1 (defined as LSK Flt3^−^, CD150^+^CD48^+^) and MPP2 (defined as LSK Flt3^−^, CD150^−^CD48^−^) from FL **(b)** or BM **(c)**, (n = 6) All error bars are means ± SEM (*p < 0.01, **p < 0.001, ***p < 0.0001). (**d)** FL or BM cells with the indicated genotype stained with Mitotracker green (total mitochondria) and Mitotracker deep red (respiring mitochondria) for 30 min and analysed by flow cytometry. (**e)** Level of reactive oxygen species (ROS) was assessed by flow cytometry using DHFCA staining on gated HSCs after incubation for 30 min at 37 °C. (n = 3) (**f)** Level of double strand breaks was assessed by flow cytometry using antibody against γ-H2AX on gated LSK (Lin^−^Sca-1^+^cKit^+^) population from FL or BM. Gray filled, isotype control; black line, control and red line, hnRNP L deleted cells (n = 3).

To gain further insight into the role of hnRNP L, we assessed cell cycle parameters and found a significant decrease in the percentage of G2/M-phase cells and a distinct increase in a sub-G1 fraction of hnRNP L deficient HSCs compared to wt HSCs (Fig. 5a). Both hnRNP L deficient fetal and adult HSCs and MPPs showed a higher frequency of Annexin V positive cells compared to wt controls (Fig. 5b,c), suggesting that hnRNP L deficient HSCs undergo apoptosis. The absence of hnRNP L also resulted in a loss of mitochondrial membrane potential in live FL and BM Lin- cells (Fig. 5d) as determined by using two mitochondria-specific labels that distinguish total (Mitotracker green) and respiring mitochondria (Mitotracker deep red). In addition, the level of reactive oxygen species (ROS) was elevated in hnRNP L deficient HSCs over controls (Fig. 5e). To assess whether apoptosis due to hnRNP L loss was primarily caused by mitochondrial dysfunction, fetal livers from VavCre^+^hnRNP L^fl/fl^ mice that also carry an anti-apoptotic H2k-Bcl-2 transgene were observed for frequencies of Annexin V positive cells. Surprisingly, over-expression of Bcl-2 did not reduce the level of Annexin V positive HSCs in VavCre^+^hnRNP L^fl/fl^ embryos (Figure S3a). Previous studies have reported a role for hnRNP L in preventing the decay of the mRNA encoding Bcl-2^38^, but we did not find evidence that the deletion of hnRNP L affected the steady state expression level of Bcl-2 mRNA (Figure S3b). This excluded Bcl-2 mRNA stability or expression level as the cause of the increased apoptosis rates seen in hnRNP L deficient, Bcl-2 over-expressing HSCs.

High levels of mitochondrial ROS production are known to potentially cause DNA damage^39^. Indeed, we observed that hnRNP L deficient LSKs expressed elevated levels of phosphorylated H2AX (γ-H2AX), an indicator of DNA double strand breaks (Fig. 5f). This suggests that a DNA damage response pathway is activated in hnRNP L deficient cells. However, treatment of hnRNP L deficient Lin^−^ cells with KU-55933, an inhibitor of ATM, VE-822, an inhibitor of ATR, or both inhibitors simultaneously did not rescue the cells from dying as assessed by Annexin V and PI staining (Figure S4). This indicates that the observed cell death in hematopoietic cells lacking hnRNP L is not the sole result of DNA damage response-induced pathways.

### Activation of p53 and p53-dependent pathways in hnRNP L null FL cells

**Figure 6:**
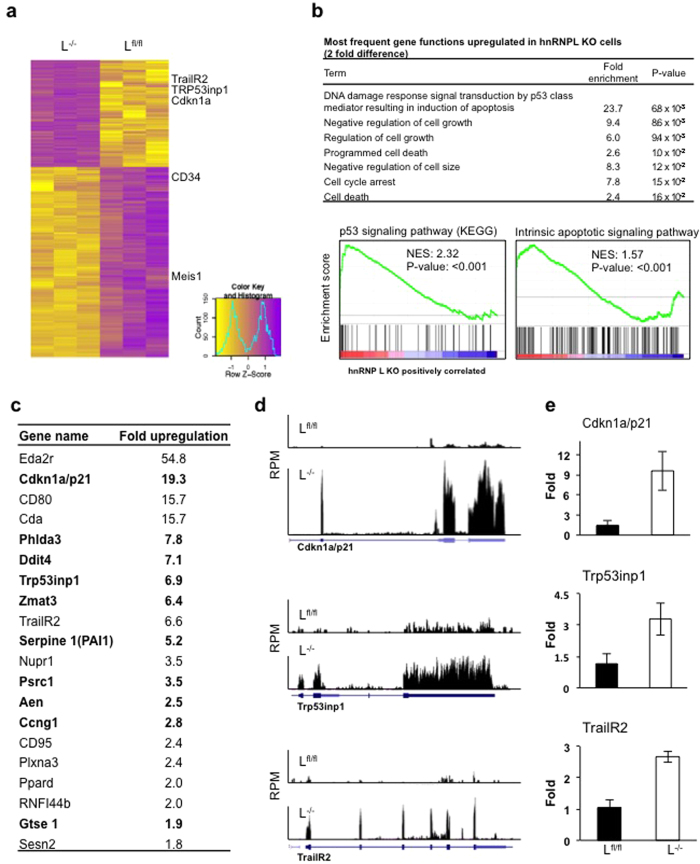
Up-regulation of p53 protein and of genes associated with p53-dependent response in hnRNP L deficient cells. (**a)** Heatmap representation of genes significantly differentially expressed (>2 fold change in expression, FDR < 0.05) between hnRNPL KO versus hnRNPL wt Lin^−^cKit^+^ samples. Genes of interest are highlighted. (**b)** Top: GO Biological processes identified as being over-represented among genes up-regulated in hnRNPL KO cells according to the DAVID software. Bottom: GSEA analysis confirming the enrichment of selected functions in the hnRNPL KO samples. Normalized Enrichment Score (NES) and nominal P-values are shown. **(c)** Fold increase in hnRNPL KO cells of selected genes of interest, as determined by RNA-Seq. Highlighted in bold are p53 target or effector genes. (**d)** Representation of RNA-Seq reads from hnRNPL WT (L ^fl/fl^) and KO (L^−/−^) Lin^−^cKit^+^ samples on the *p21* (CDKN1a), *Trp53inp1* and *TrailR2* loci. The y-axis shows reads per million aligned reads (RPM). (**e**) Validation of overexpression of selected genes from b) using RT-qPCR on flow sorted Lin^−^cKit^+^ cells from wt or hnRNP L deficient mice normalized relative to expression of Gapdh.

To gain more insight into the molecular defects of hnRNP L deficient HSCs, we undertook a genome-wide analysis of mRNA expression and splicing through next-generation RNA sequencing using three independent preparations of wt or VavCre^+^hnRNP L^fl/fl^ E14.5 Lin^−^cKit^+^ FL cells (Fig. 6a). We first quantified splicing differences by mapping sequenced reads against all known annotated splice junction combinations within a gene locus, regardless of prior evidence of their usage *in vivo*. Surprisingly, this analysis did not reveal any significant differences in splicing between the wt and hnRNP L deficient cells. We also quantified variations in splicing through Differential Exon Usage (DEU) analysis, which only highlighted 38 genes having at least one exon with greater than 2-fold change in use (Table S2), after correction for transcript level changes. Functional analysis did not reveal any biological process significantly associated with these genes (Table S3). Notably, genes involved in apoptosis, such as Trp53, TrailR2 or FAS showed no significant DEU (Figure S5).

We next examined differentially expressed genes and observed that at a 2-fold threshold of up- or down-regulation, 470 genes were up-regulated and 88 genes were down-regulated in hnRNPL deficient cells. The genes down-regulated in their expression in hnRNP L deficient versus wt cells belonged mostly to GO categories such as hematopoietic differentiation and cell activation (Figure S5a,b). Notably, many genes important for HSC functions such as *Gfi1, Runx1, Meis1, PU.1, CD34, Notch1* and *Cxcr4*, but also cytokine receptor genes, were affected (Figure S5c). The down-regulation of their expression in the absence of hnRNP L was confirmed by RT-qPCR (Figure S5d).

The most significant enrichment for the biological functions of up-regulated genes was found in a GO biological process related to p53 (Fig. 6b) including many p53-target or -effector genes such as *Cdkn1a, Tp53inp1, Zmat3* or *Ccng1*, but also other genes involved in cell death such as *CD95* and *TrailR2* (Fig. 6c,d). Further functional assessment using GSEA confirmed enrichment of the p53 signaling and intrinsic apoptotic signaling pathways (Fig. 6b). To confirm that the RNA-Seq results represent actual expression differences in the FL subset, we validated the expression changes of three of these genes (*Cdkn1a, TrailR2* and *Tp53inp1*) by RT-qPCR using RNA from sorted LSK cells from hnRNP L deficient FL (Fig. 6e). Finally, analysis of p53 targets and effectors deregulated in their expression in hnRNP L deficient cells confirmed that several p53 dependent pathways are affected by hnRNP L ablation (Figure S7). In agreement with this, we observed higher than control levels of p53 protein in hnRNP L deficient FL cells (Figure S8a), which was not accompanied by increased expression or half-life of p53 mRNA (Figure S8b,c), suggesting that the deletion of hnRNP L does not affect p53 transcription directly.

To test whether the activation of a p53 pathway was responsible for the loss of cell functionality observed in hnRNP L deficient HSCs, we generated Vav-Cre, hnRNP L^fl/fl^, p53^−/−^ deficient mice. Frequencies or numbers of LSK cells in FLs of hnRNP L/p53 deficient animals were not significantly rescued (Figure S9a,b) and their ability to differentiate in an OP9 culture was not restored (Figure S9c), suggesting that other, p53-independent, cell death pathways must be active in hnRNP L deficient cells.

### hnRNP L regulates TrailR2 expression and loss of hnRNP L induces cell death via caspase-dependent pathways

**Figure 7:**
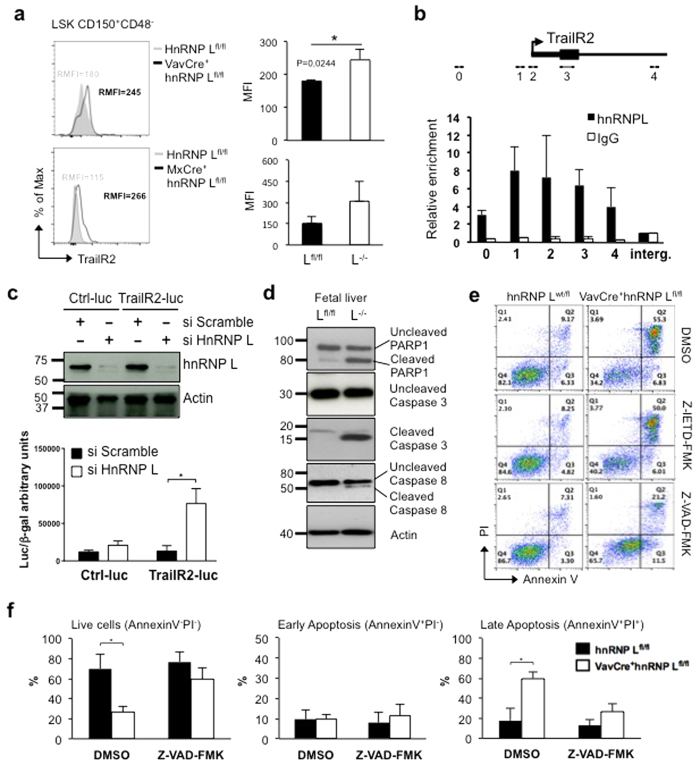
Absence of hnRNP L results in activation of cell death pathways and eventually caspase-dependent cell death. (**a)** TrailR2 expression was tested by flow cytometry on HSCs from FL of hnRNP L^fl/fl^ or VavCre^+^hnRNP L^fl/fl^ embryos and BM of hnRNP L^fl/fl^ or MxCre^+^hnRNP L^fl/fl^ mice. (**b)** Relative enrichment of hnRNP L over the *TrailR2* promoter and intergenic control (Interg.) in 3T3 cells by ChIP-qPCR. Error bars represent standard deviation (n = 3). Schematic representation of primer locations over the *TrailR2* gene locus. Arrow indicates the transcription start site (TSS) of *TrailR2*, medium thick line is the 5′ UTR, thickest line is exon 1 and the thinnest line is intron 1. (**c)** Luciferase assay for TrailR2 promoter region. HEK293T cells were transfected initially with scrambled or hnRNP L specific siRNA before transfection with the TrailR2 promoter reporter region or a control reporter construct. Luciferase activity was normalized to beta-galactosidase. Error bars represent the standard deviation (n = 3). P-values were calculated using the Student’s t-test. **(d)** Western blot analysis of PARP1 cleavage, Caspase-3 cleavage and Caspase-8 cleavage in total FL cells from hnRNP L wt embryos (L^fl/fl^) and hnRNP L deleted embryos (L^−/−^). (**e)** Flow cytometry analysis of E14.5 FL lineage negative cells from indicated genotypes treated with Caspase-8 inhibitor (Z-IETD-FMK; 100 μM), Pan-Caspase inhibitor (Z-VAD-FMK; 100 μM) or DMSO as a negative control for 24 hours in culture before Annexin V and PI levels were measured. (n = 6) (**f)** Bar graph showing the significant quantification of the treated cells in e). All error bars are means ± SEM (*p < 0.01).

Death receptors CD95/Apo1/Fas and TrailR2 were found to be up-regulated in hnRNP L deficient cells (Fig. 6d–f). Although HSCs usually do not express CD95^40^, staining of hnRNP L deficient HSCs from FL or BM revealed an abnormally high expression level of this death receptor compared to control cells (Figure S10). Similarly, HSCs from the FL or BM lacking hnRNP L were found to have higher levels of TrailR2 than wt control HSCs (Fig. 7a). To explore the possibility that hnRNP L could function as a transcriptional regulator of the *TrailR2 gene*, we tested for hnRNP L binding by ChIP-qPCR over the *TrailR2* locus. Data obtained with primers that cover the promoter region of *TrailR2* indicate a direct or indirect occupation by hnRNP L principally at the transcription start site and within the first exon (Fig. 7b), suggesting that hnRNP L participates in the control of TrailR2 expression. To directly assess hnRNP L action on *TrailR2* expression, a luciferase reporter construct carrying the *TrailR2* promoter was transfected into HEK293 cells either expressing normal or reduced protein levels of hnRNPL upon transfection of a specific siRNA against hnRNP L (Fig. 7c, upper panel). Lower levels of hnRNP L significantly increased *TrailR2* promoter activity (Fig. 7c, lower panel), suggesting that hnRNP L restricts *TrailR2* transcription by binding to its promoter region.

Signaling through cell death receptors such as TrailR2 and Fas is known to induce cleavage of Caspase-8 protein and further downstream effectors. Lin^−^cKit^+^ FL cells from hnRNP L deficient mice showed such cleaved forms of PARP1, Caspases-3 and -8 (Fig. 7d), suggesting that active signaling through cell death receptors takes place in hnRNP L deficient cells. Treatment of E14.5 hnRNP L deficient FL Lin^−^ cells for 24 hours with Caspase-8 specific inhibitor (Z-IETD-fmk), however, did not significantly reduce the number of Annexin V and PI positive cells (Fig. 7e). However, treatment with a Pan-Caspase inhibitor (Z-VAD-fmk) for 24 hours significantly reduced cell death from approximately 60% to 25% (Fig. 7e,f). This indicates that hematopoietic cell death due to an absence of hnRNP L is primarily mediated through Caspase-dependent pathways.

## Discussion

Both the regulation of gene transcription and RNA metabolism play important roles in controlling the maintenance of HSC self-renewal, survival and differentiation^16^,^17^,^18^,^20^. In this study, we set out to explore the role of the RNA binding protein and splicing factor hnRNP L and we present evidence that this factor is indeed critical for the functional integrity of HSCs and thus for normal hematopoiesis. The deletion of hnRNP L leads to the disruption of fetal and adult hematopoiesis and causes hematopoietic failure characterized by neutropenia, thrombocytopenia, anemia and premature death in adult mice. In addition, hnRNP L deficient, phenotypically defined HSCs were unable to elicit hematopoietic differentiation in transplanted hosts. These findings clearly establish hnRNP L as a novel regulator of hematopoiesis and an essential factor for hematopoietic stem cells. Unexpectedly, the data from our experiments suggest that hnRNP L exerts this function in hematopoietic stem- and progenitors, at least in part, as a regulator of transcription rather than acting as a splicing factor, a well described function of this protein in many other cell types and cell lines.

### hnRNP L regulates cellularity of HSCs and is essential for their functional integrity

When phenotypically defined by staining for surface markers, HSCs seem to have differential requirements for hnRNP L depending on their developmental stage, since their numbers are clearly reduced in adult BM of hnRNP L deficient mice, but are found at rather normal levels in hnRNP L deficient FLs. One possible explanation for this may be the fact that FL HSCs have a higher proliferative capacity than adult HSCs, which are almost all quiescent^14^,^15^. We cannot rule out that this is due to the use of different Cre alleles. However, both fetal and adult hnRNP L deficient HSCs have lost all critical stem cell functions and, most importantly, their capacity to induce multi-lineage differentiation, shown by experiments such as differentiation on OP9 feeder layers and transplantation assays, very likely due to their high propensity to undergo cell death. In particular, our findings that hnRNP L deficient adult mice have less HSCs than wt mice can be explained by a higher rate of cell death, which was indeed confirmed by Annexin V and PI staining of progenitor cells suggesting that hnRNP L is required for their survival. Our RNA-Seq data confirm this notion since they show up-regulation of many cell death associated genes in hnRNP L deficient cells among them p53 effectors and genes for the death receptors and TNF family members CD95 and TrailR2.

### Mitochondrial stress is activated in hnRNP L deficient cells

Our finding that loss of hnRNP L is associated with mitochondrial dysfunction is in agreement with previous reports that hnRNP proteins are involved both in nuclear RNA transcription and processing and also play important roles in mitochondria. High-throughput sequencing (CLIP-seq) in T cells to find RNA binding sites of hnRNP L revealed a significant number of transcripts aligned to mitochondrial DNA^41^. Additionally, hnRNP K, another member of the family of RNA-binding proteins, has been shown to localize to the mitochondria and been co-purified with mitochondrial RNA^42^,^43^. It is thus conceivable that hnRNP L is essential for transcription and/or RNA processing in the ‘powerhouse of the cell’ and its loss leads to mitochondrial dysfunction. Compromised mitochondrial gene expression has previously been shown to elevate ROS production and reduce life span^44^. This is similar to the increased ROS levels we detected in hnRNP L null HSCs, which is a known activator of the DNA damage and p53-dependent pathways. However, mitochondrial stress is likely not the sole cause of cell death upon hnRNP L loss since over-expression of the anti-apoptotic protein Bcl-2, which can counteract apoptosis due to mitochondrial dysfunction, did not restore cell survival in hnRNP L deficient cells.

### DNA damage response pathways are activated in hnRNP L deficient cells

Both fetal and adult hnRNP L deficient HSCs showed increased levels of phosphorylated H2AX, which is one of the very first events subsequent to DNA damage^45^. It is possible that the increased levels of ROS are causing DNA damage and subsequently also the increase in H2AX phosphorylation, but it cannot be excluded that loss of hnRNP L also leads to genomic instability, replication stress and DNA damage as was reported for the hnRNP Npl3^46^. Our RNA-Seq data are also in agreement with an ongoing DNA damage response in hnRNP L deficient cells since the up-regulation of p53 effectors and other genes that partake in a DNA damage response pathway is detected when hnRNP L is absent. However, the inability of ATM/ATR inhibitors to restore survival of hnRNP L null cells indicates that single and double stranded DNA breaks are not the only driver of cell death in the absence of hnRNP L.

Several data indicate that p53 plays a central role in the accelerated cell death of hnRNP L deficient HSCs. Most strikingly, the expression of p53 protein was drastically enhanced in hnRNP L deficient fetal liver cells. These high levels of p53 protein were not caused by a transcriptional up-regulation of the p53 gene as shown by RT-qPCR analysis nor by an altered half-life of the p53 mRNA. It is possible that hnRNP L regulates the translation efficiency of p53 possibly through direct binding to its mRNA as has been reported previously^41^,^47^. Alternatively, this activation of p53 may simply be the consequence of the ongoing DNA damage response in hnRNP L deficient cells. Both possibilities, however, would offer an explanation for the up-regulation of many DNA response genes and p53 target genes in hnRNP L deficient cells seen in the RNA-Seq analysis. Importantly though, p53 knockout was unable to rescue hnRNP L deficiency-induced cell death. This suggests that the activation of p53 is not the main cause of cell death in hnRNP L deficient cells but likely rather a consequence of DNA damage caused by elevated ROS levels or other yet unidentified stress signals in hnRNP L deficient cells.

### hnRNP L controls the expression of death receptors and caspase-dependent cell death

Our RNA-Seq data indicate that both CD95 and TrailR2 (also called DR5) mRNAs are up-regulated in hnRNP L deficient cells, suggesting that hnRNP L affects the transcriptional regulation of these genes. Indeed, our findings that hnRNP L is localized at the *Trailr2* promoter region and that a *TrailR2* promoter driven reporter gene is activated upon hnRNP L knockdown in cell lines clearly support this notion. It is therefore likely that hnRNP L controls death receptor signaling which is known to initiate several forms of cell death including apoptosis and necroptosis. Our observation that the effectors of CD95/Fas/Apo1 and TrailR2, Caspases-3 and -8, as well as PARP1 are cleaved in hnRNP L deficient hematopoietic progenitor cells clearly indicates active death receptor signaling in hnRNP L deficient cells. Our findings that hnRNP L deficient cells show mitochondrial dysfunction, increased levels of ROS and activated p53 pathways would be in agreement with this, since death receptor signaling has previously been shown to cause loss of mitochondrial polarity^48^,^49^,^50^. Finally, our finding that Bcl-2 overexpression does not restore cell survival in hnRNP L deficient HSCs supports this notion, because death receptor/caspase-8 cell death pathways can be initiated independently of Bcl-2^51^.

The most unequivocal evidence that increased Caspase-dependent death receptor signaling is responsible for the cell death seen when hnRNP L is deleted comes from our experiment with pan-Caspase inhibitors. This treatment significantly restores the viability of hnRNP L deficient cells in culture, strongly suggesting that hnRNP L controls Caspase-dependent death pathways in hematopoietic stem and progenitors cells. This experiment also indicated that the form of cell death that is controlled by hnRNP L may be necroptosis rather than apoptosis, since almost all dying or dead cells from hnRNP L deficient mice have incorporated PI. Although this has to be confirmed by additional experiment, these data are in accordance with previous reports describing Caspase-dependent Trail- and FasL-induced necroptosis^52^,^53^ and other Caspase-dependent necrosis-like cell death pathways^54^,^55^.

### Transcription, but not alternative splicing, is deregulated in hnRNP L null HSCs

It was surprising that our RNA-Seq analysis of hnRNP L deficient FL cells revealed altered regulation of mRNA expression levels, but no differences in alternative splicing, since hnRNP L is a splicing factor and clear evidence support its role in the alternative splicing of many genes, most notably CD45^24^. In addition, we have previously reported that conditional deletion of hnRNP L using the same animals as in this study affects alternative splicing in T-cells notably the CD45 pre-mRNA^26^ confirming this well-established function of hnRNP L. However, the biological functions of hnRNP L and their cell type specificity have not been fully clarified yet and it is possible that hnRNP L is also involved in transcriptional regulation. A few reports have already provided evidence for such a role; for instance, hnRNP L was found to be required for transcription of the TNFalpha gene^46^ and has also been associated with the mediator complex associated with transcription initiation and elongation^56^,^57^. Our data with the TrailR2 gene lend further support to the idea that hnRNP L also regulates transcription and not only alternative splicing. The published reports, along with our results, suggest that hnRNP L may be involved in several different aspects of regulating mRNA transcription and splicing, which may be highly context dependent or restricted to specific cell types. It is however also possible that fully deleted HSCs are rapidly lost and that cells with incomplete deletions of hnRNP L containing residual hnRNP L protein remain in the analysis and obscure the RNA-Seq analysis making a detection of alternative splicing events difficult. This would not be surprising given that several cell death pathways are activated and numbers of functional HSCs are strongly decreased in hnRNP L deficient animals. Overall, our findings provide compelling evidence that hnRNP L is a novel regulator essential for survival and functional integrity of HSCs and thereby for hematopoietic differentiation, acting by controlling Caspase-dependent cell death pathways.

## Additional Information

**How to cite this article**: Gaudreau, M.-C. *et al. Heterogeneous Nuclear Ribonucleoprotein L* is required for the survival and functional integrity of murine hematopoietic stem cells. *Sci. Rep.* **6**, 27379; doi: 10.1038/srep27379 (2016).

## Supplementary Material

Supplementary Information
