# Supplementary material for: Heterogeneous Nuclear Ribonucleoprotein L is required for the survival and functional integrity of murine hematopoietic stem cells
Source: Sci Rep. 2016 Jun 7;6:27379. doi: 10.1038/srep27379 (PMC4895350; doi:10.1038/srep27379)
Supplement: Supplementary Information [file srep27379-s1.pdf]

Supplementary information

Heterogeneous Nuclear Ribonucleoprotein L is required for the survival and functional integrity of murine hematopoietic stem cells

Marie-Claude Gaudreau[1,2,+], Damien Grapton[1,2,+], Anne Helness[1,3,+], Charles Vadnais[1,2], Jennifer Fraszczak[1,2], Peiman Shooshtarizadeh[1], Brian Wilhelm[4], François Robert[1,5], Florian Heyd[6], Tarik Möröy[1,2,*]

[+] these authors contributed equally

[1]Institut de recherches cliniques de Montréal (IRCM)

[2]Département de microbiologie, infectiologie et immunologie, Université de Montréal, Montréal, Canada,

[3]Department of Experimental Medicine, McGill University, Montréal, Canada,

[4]Institut de recherche en immunologie et cancerologie, Université de Montréal,

[5]Département de médecine, Faculté de médecine, Université de Montréal, Montréal, Canada,

[6]Institut für Chemie und Biochemie, Freie Universität Berlin, Berlin, Germany

[*]Corresponding author: Tarik.Moroy@ircm.qc.ca

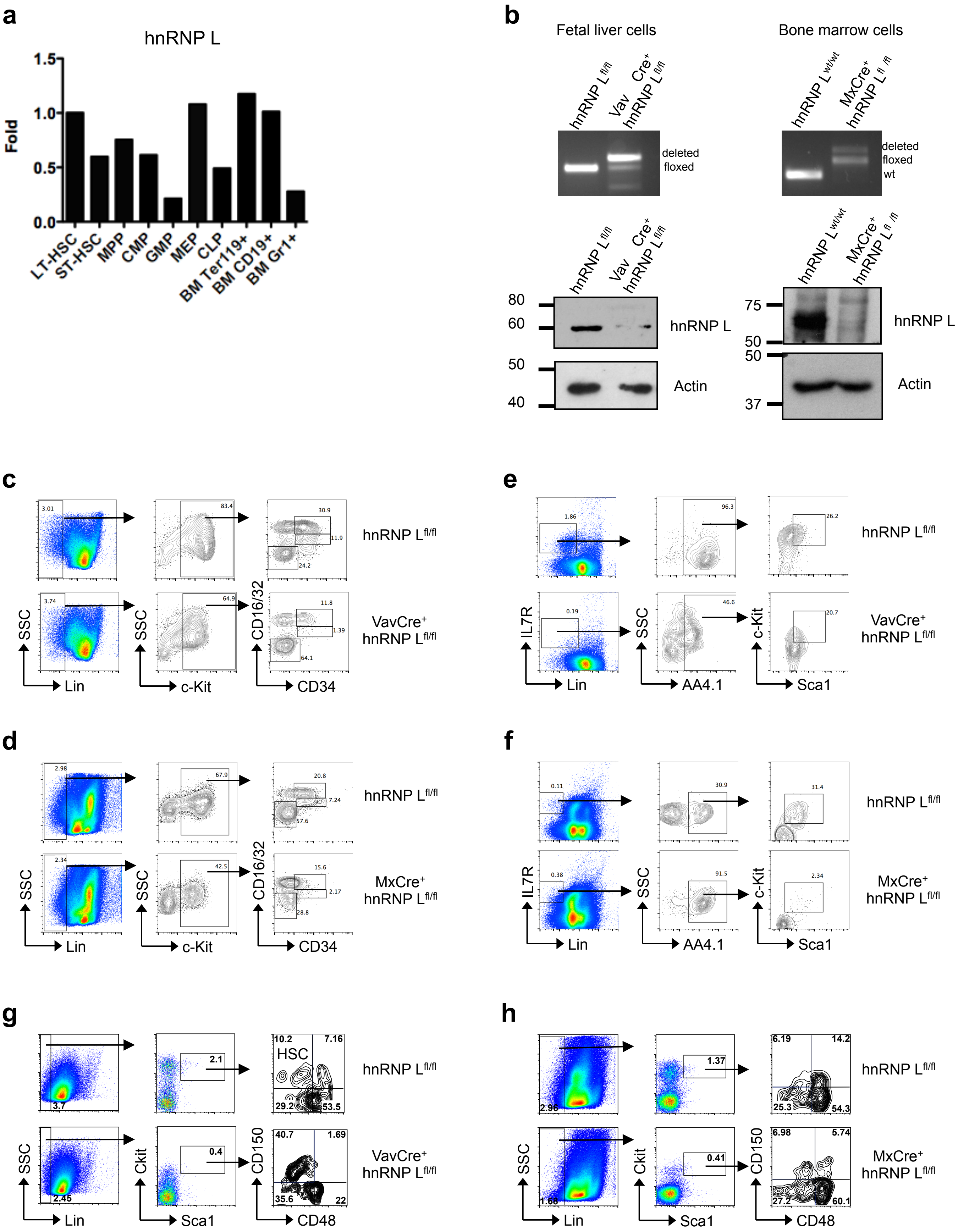
a
hnRNP L
Fold
1.5
1.0
0.5
0.0
LT-HSC
ST-HSC
MPP
CMP
GMP
MEP
CLP
BM Ter119+
BM CD19+
BM Gr1+
b
Fetal liver cells
Bone marrow cells
deleted
floxed
wt
hnRNP L
Actin
c
d
e
f
g
h
SSC
Lin
c-Kit
CD16/32
CD34
IL7R
AA4.1
c-Kit
Sca1
Ckit
CD150
CD48
HSC
VavCre+ hnRNP L fl/fl
MxCre+ hnRNP L fl/fl
hnRNP L fl/fl

Supplemental Figure S1:

a) RT-qPCR analysis with RNA isolated from the indicated bone marrow fractions show relative levels of hnRNP L expression. **b)** PCR on genomic DNA from FL (upper left panel) or BM (upper right panel) of the indicated mice shows the deletion of floxed parts of the hnRNP L gene in the presence of a VavCre transgene (upper left panel) or an MxCre transgene (upper right panel). Western blot analysis of hnRNP L protein levels in FL (lower left panel) or BM (lower right panel) of the indicated mice. **c) & d)** Examples for the gating strategy used to detect myeloid and erythroid progenitors (MEP, CMP and GMP) in the FL (c) and in the BM (d). The lineage cocktail for these experiments contained antibodies against B220, Ter119, CD3, CD11b, Gr1, NK1.1, CD49b, CD8 and Sca1. **e) & f)** Examples for the gating strategy used to detect lymphoid progenitors (CLP) in the FL (e) and in the BM (f). **g) & h)** Representative plots showing the gating strategy used to detect HSCs and progenitors (MPP1 and MPP2) in FL (e) and BM (f).

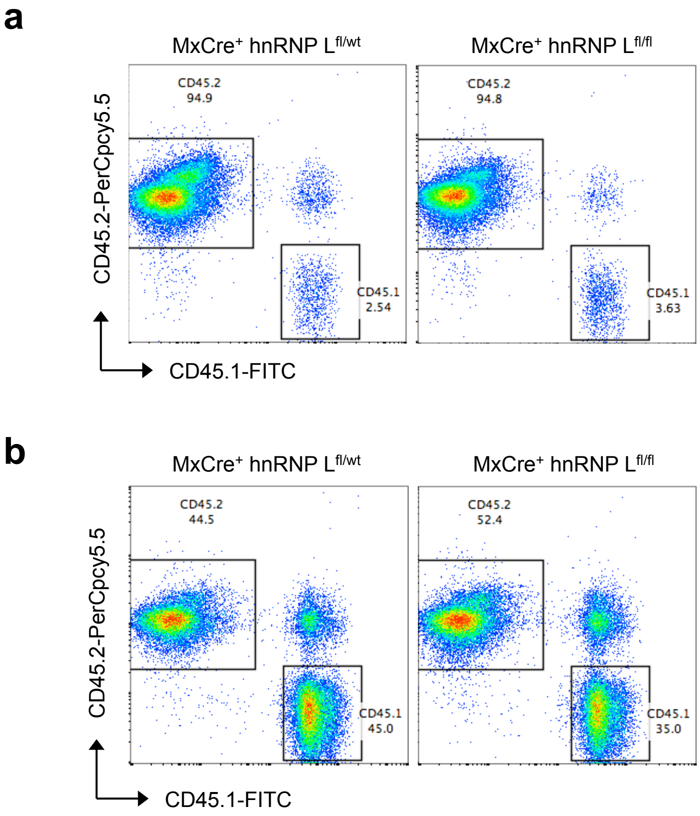

Supplemental Figure S2:

Flow cytometry analysis for donor chimerism of lymphocytes in the blood of irradiated wt CD45.1+ mice intravenously inoculated **a)** non-competitively or **b)** competitively (with total BM cells from wt CD45.1+ mice at a 1:1 ratio) with either total BM cells from MxCre $^{+}$hnRNPL $^{fl/fl}$ or MrCre^{+}hnRNP L $^{wt/fl}$ (CD45.2^{+}) and analyzed 10 weeks after transplantation.

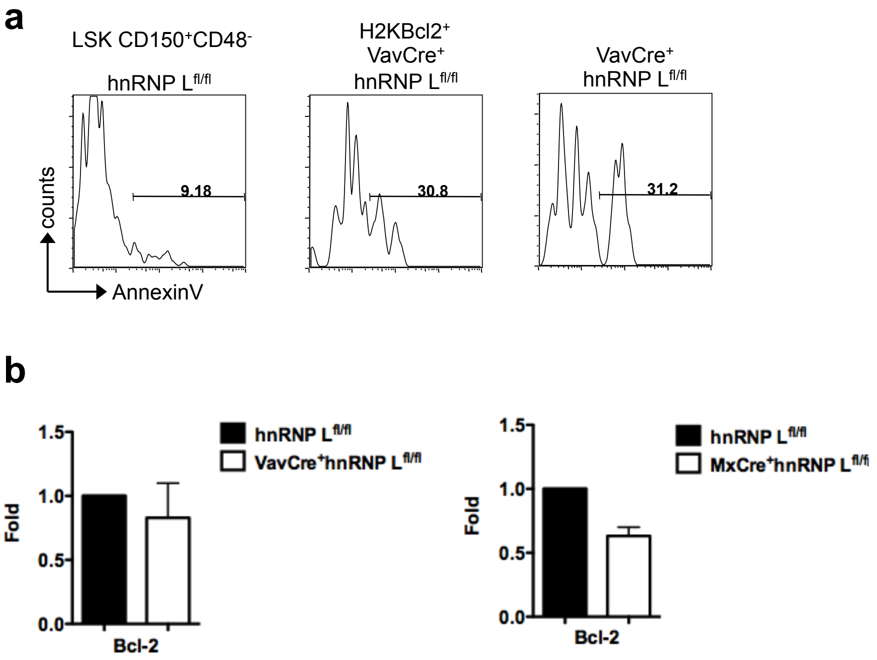

Supplemental Figure S3:

a) Flow cytometric analysis of AnnexinV positive HSCs fetal livers from embryos with indicated genotype. **b)** Quantification of Bcl-2 mRNA expression by real-time PCR on lineage depleted FL or BM cells from embryos or adult mice, respectively, with the indicated genotype. Data are shown as fold induction relative to values obtained for wt control. Average of triplicate values ± SEM are shown.

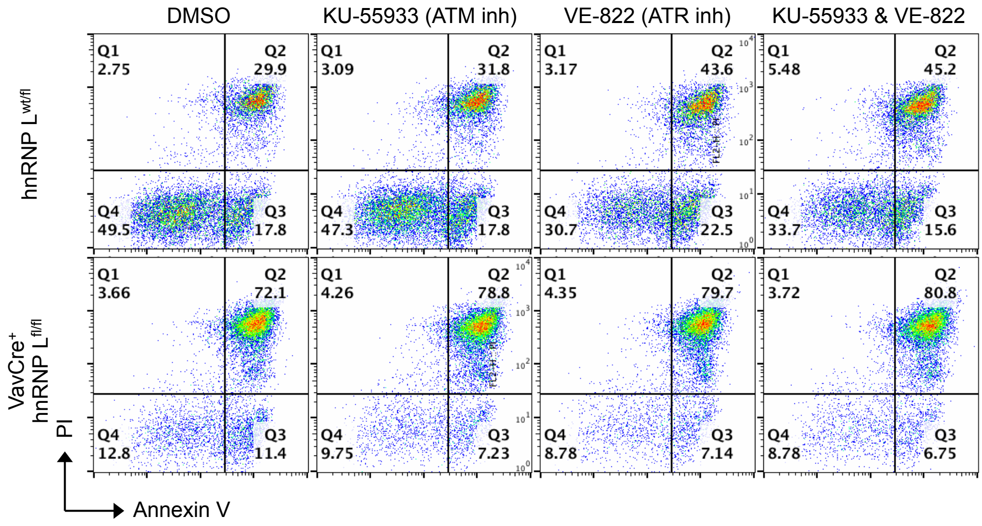

Supplemental Figure S4:

Flow cytometry analysis of E14.5 FL lineage negative cells from indicated genotypes treated with ATMi (KU-55933; 1nM), ATRi (VE-822; 1nM) or both inhibitors or DMSO as a negative control for 24 hours in culture before AnnexinV and PI levels were measured.

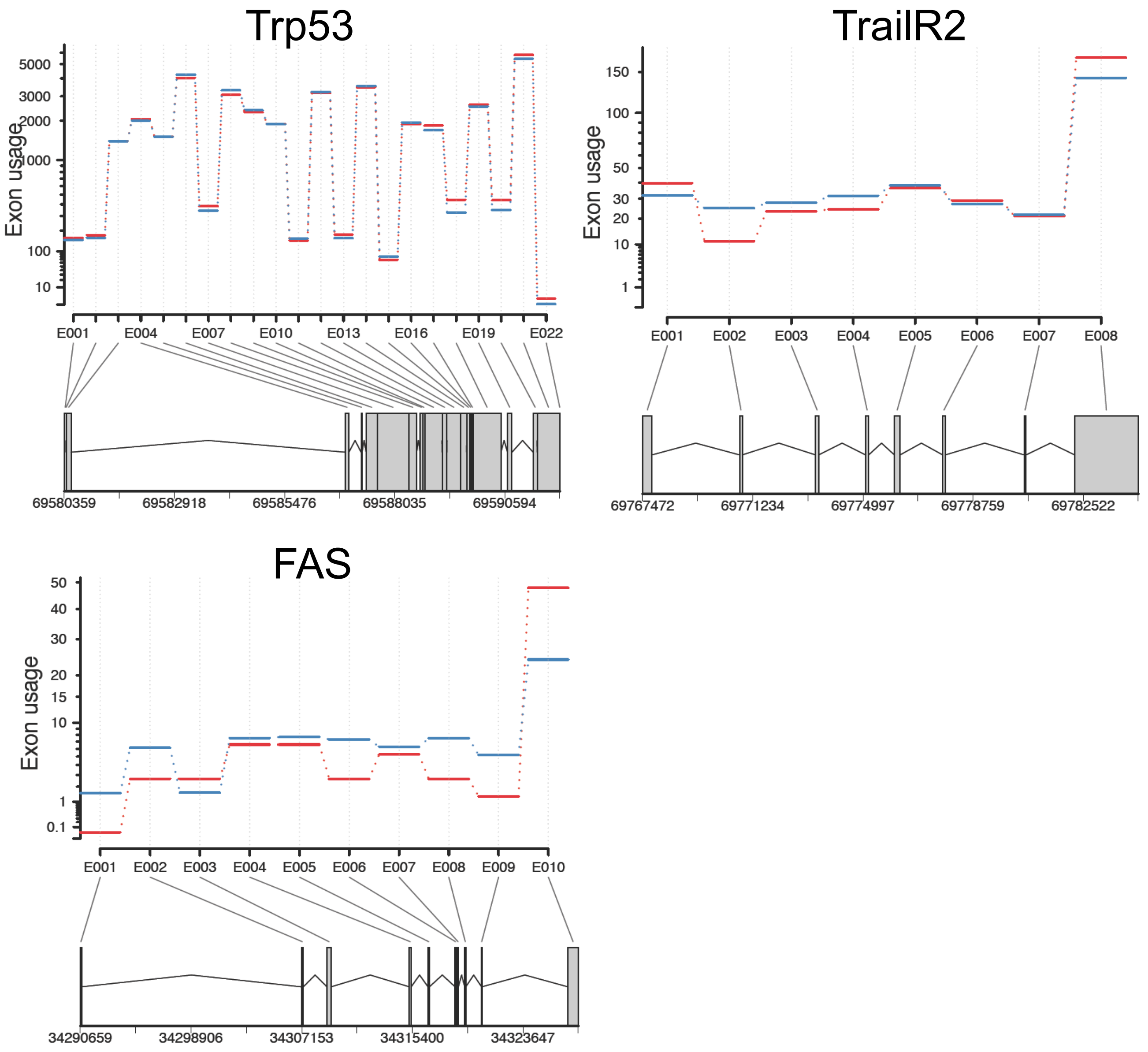

Supplemental Figure S5:
Visual representation of Differential Exon Usage for selected genes. Exon use levels in hnRNPL wild-type samples are shown as red lines, hnRNPL KO samples as blue lines. None of the exons shown for the selected genes display statistically significant differences.

a

Most frequent gene functions downregulated in hnRNPL KO cells (2 fold difference)

Term	Fold enrichment	p-value
Cell activation	5.1	7.3×10^{-10}
Leukocyte activation	4.5	5.2×10^{-7}
Lymphocyte activation	4.6	2.1×10^{-6}
Immune response	2.9	5.3×10^{-6}
T cell activation	5.7	$8.4 \times 10^{--6}$
Lymphocyte differentiation	5.3	4.3×10^{-5}
Leukocyte differentiation	4.6	5.6×10^{-5}
T cell differentiation	6.5	6.9×10^{-5}

b

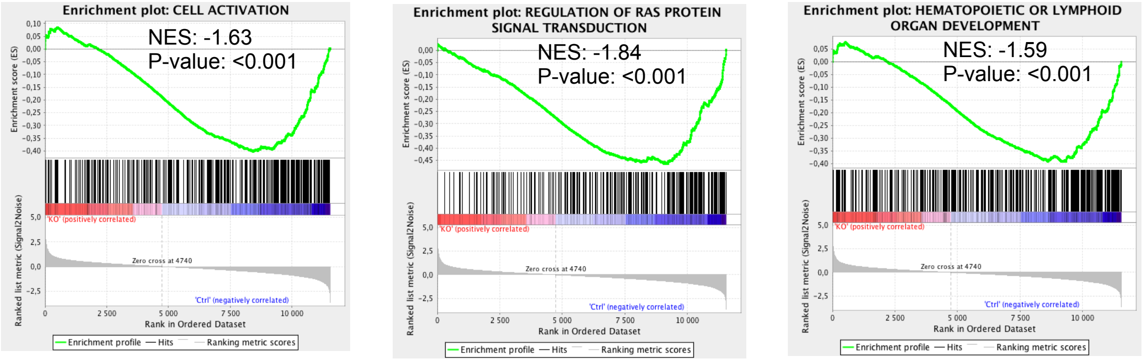

c

**Gene name**	**Fold down-regulation**
M-CSFR	0.11
Mpeg1	0.11
Tifab	0.13
Satb1	0.14
Clec12a	0.14
Ifitm1	0.15
Ifi203	0.14
Hp	0.16
Parp8	0.16
Lsp1	0.16
Cathepsin G	0.17
CD93	0.17
Gda	0.17
Prtn3	0.18
Rassf4	0.18
Rgs2	0.18
Atp8b4	0.18
Mtus1	0.18
Rab44	0.18
Gpr97	0.18
Mpo	0.18
Serpinb1a	0.19
Cxcr4	0.20
Il6ra	0.20
Glipr1	0.21
CD34	0.22
Gfi1	0.23
G-CSFR	0.21
Igfbp4	0.26
PU.1	0.24
Runx1	0.52

d

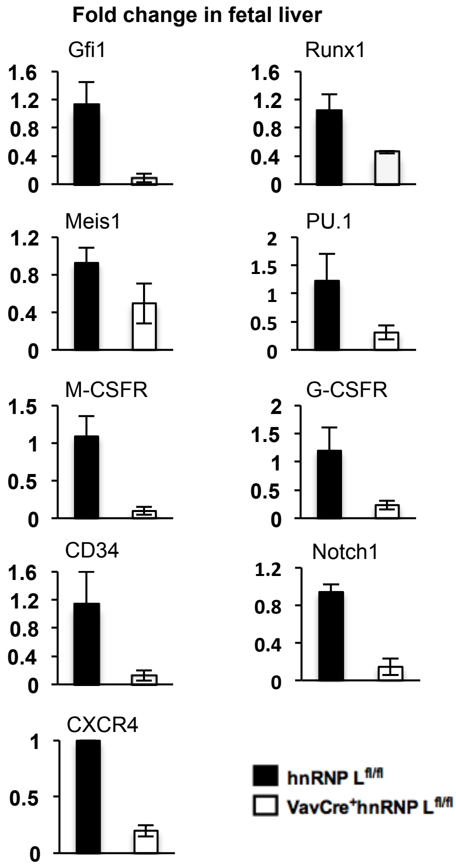

Supplemental Figure S6:

a) GO Biological processes identified as being over-represented among genes down-regulated in hnRNPL KO $Lin^{-}cKit^{+}$ cells according to the DAVID software. **b)** GSEA analysis confirming the enrichment of selected functions in the hnRNPL WT samples. Normalized Enrichment Score (NES) and nominal P-values are shown. **c)** Fold decreases in hnRNPL KO cells of selected genes of interest, as determined by RNA-seq. **d)** RT-qPCR analysis of selected genes expression relative to Gapdh with RNA isolated from the indicated $Lin^{-}cKit^{+}$ fetal liver cells.

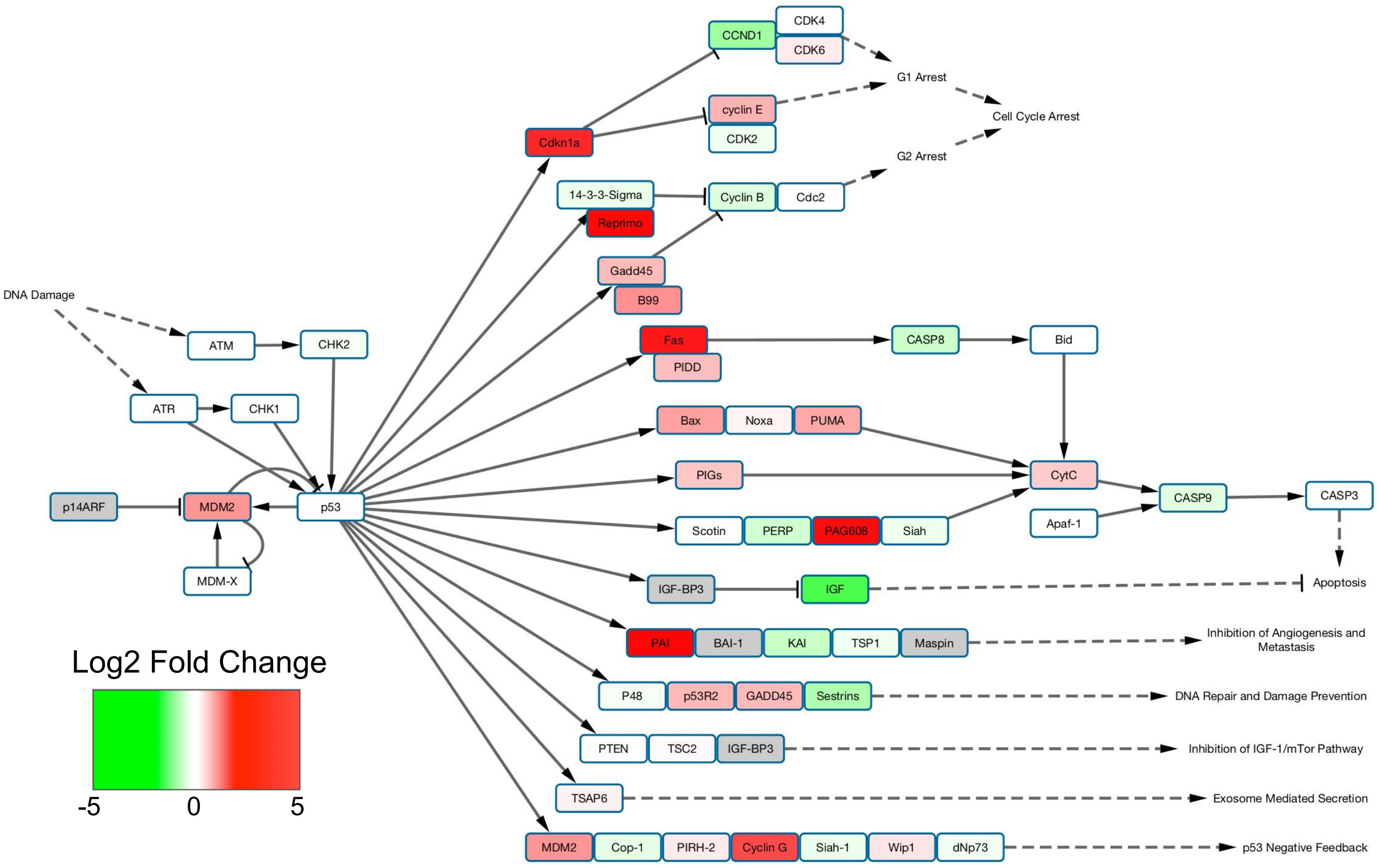

Supplemental Figure S7:
Visual representation of the p53-signalling pathway (based on KEGG entry mmu04115) using a colour coded representation of changes in expression in hnRNPL KO cells compared to wild-type cells. Green: down-regulation, Red: up-regulation, White: no change, Grey: no data. All values are expressed as log2 fold changes. Generated using Cytoscape v3.1.0.

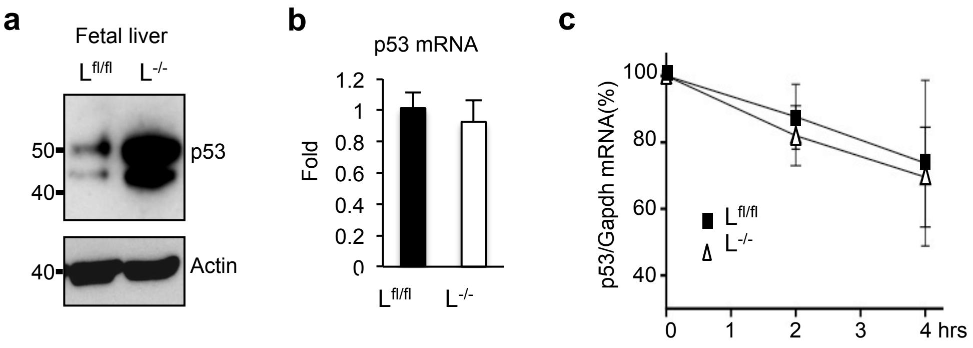

Supplemental Figure S8:

a) Western blot analysis of p53 levels in total FL cells from embryos with the indicated genotype. **b)** RT-qPCR analysis of p53 mRNA expression relative to Gapdh in sorted $Lin^{-}cKit^{+}$ FL cells from embryos with the indicated genotype **c)** Sorted $Lin^{-}cKit^{+}$ FL cells from embryos with the indicated genotype were treated with 5 µg/mL actinomycin D and harvested at the indicated time points. The percentage of p53 mRNA remaining at each time point was assessed by RT-qPCR and normalized to Gapdh.

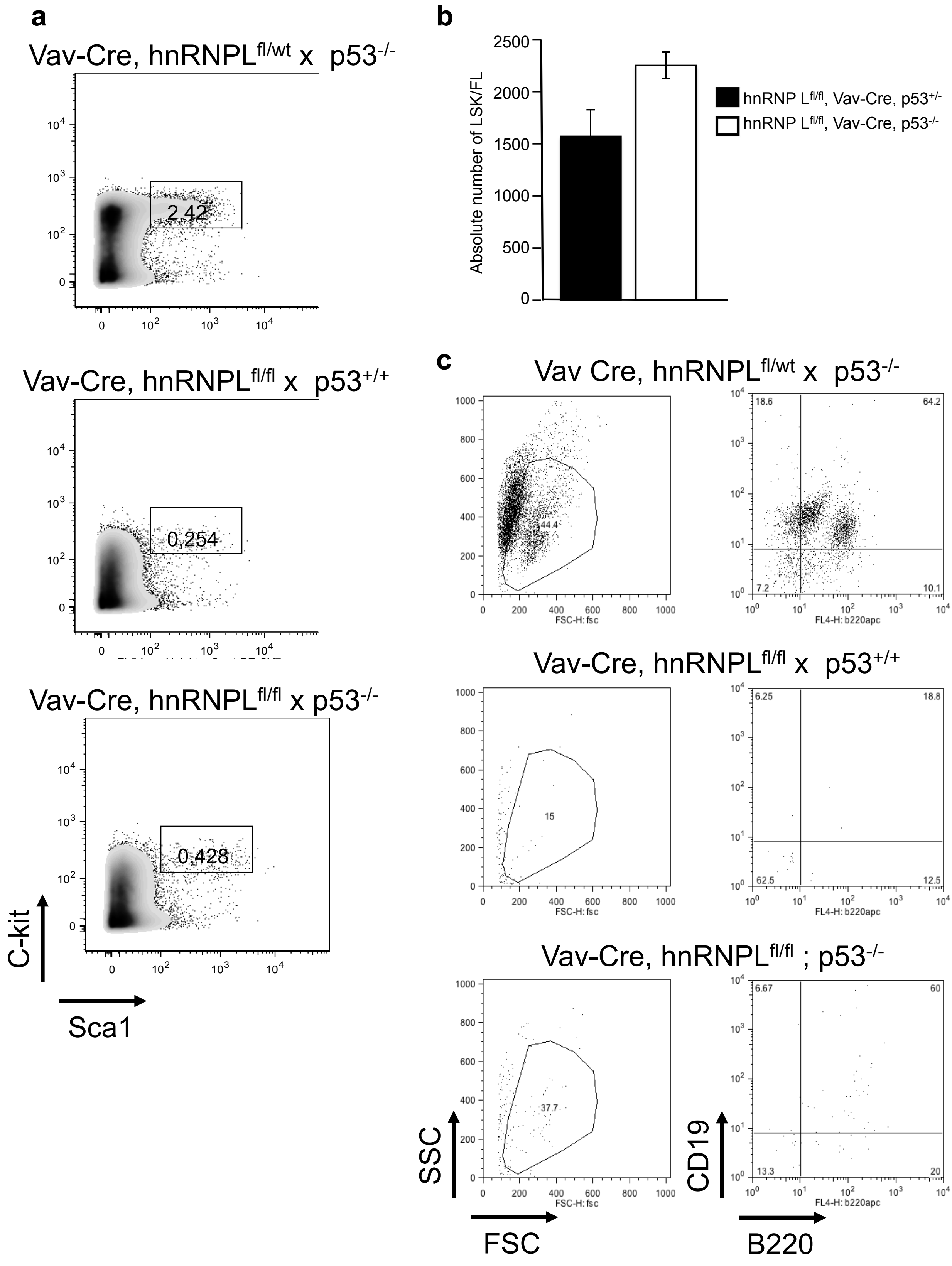

a
Vav-Cre, hnRNPL$^{fl/wt}$ x p53$^{-/-}$
2.42
Vav-Cre, hnRNPL$^{fl/fl}$ x p53$^{+/+}$
0,254
Vav-Cre, hnRNPL$^{fl/fl}$ x p53$^{-/-}$
0,428
C-kit
Sca1
b
Absolute number of LSK/FL
hnRNP L$^{fl/fl}$, Vav-Cre, p53$^{+/-}$
hnRNP L$^{fl/fl}$, Vav-Cre, p53$^{-/-}$
c
Vav Cre, hnRNPL$^{fl/wt}$ x p53$^{-/-}$
44.4
18.6
64.2
7.2
10.1
Vav-Cre, hnRNPL$^{fl/fl}$ x p53$^{+/+}$
15
6.25
18.8
62.5
12.5
Vav-Cre, hnRNPL$^{fl/fl}$; p53$^{-/-}$
37.7
6.67
60
13.3
20
FSC-H: fsc
FL4-H: b220apc
SSC
FSC
CD19
B220

Supplemental Figure S9:

a) Representative flow cytometry analysis of LSK cells in the indicated mice. **b)** Graph showing the absolute number of LSK cells. **c)** 5000 fetal liver Lin- cells were sorted from E14.5 embryos and plated on OP9-DL4 stromal cells. OP9-DL4 cultures containing 10μg/ml Flt3L and 10μg/ml IL-7. Cells were analyzed for the expression of B-cells markers 7 and 14 days later.

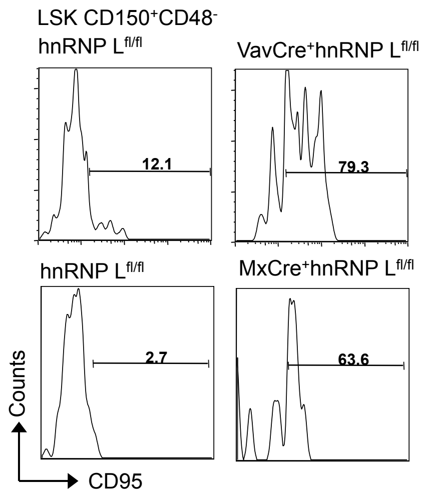

Supplemental Figure S10:
CD95 expression was tested by flow cytometry on HSCs from fetal livers of hnRNP $L^{fl/fl}$ or VavCre^{+}hnRNP $L^{fl/fl}$ embryos (upper part) and from bone marrow of hnRNP $L^{fl/fl}$ or MxCre^{+}hnRNP $L^{fl/fl}$ mice (lower part).

Figure 7c:

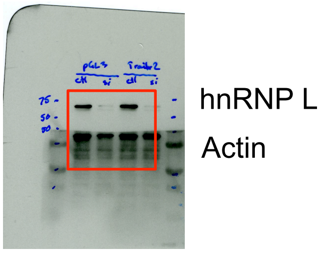

Figure 7d:

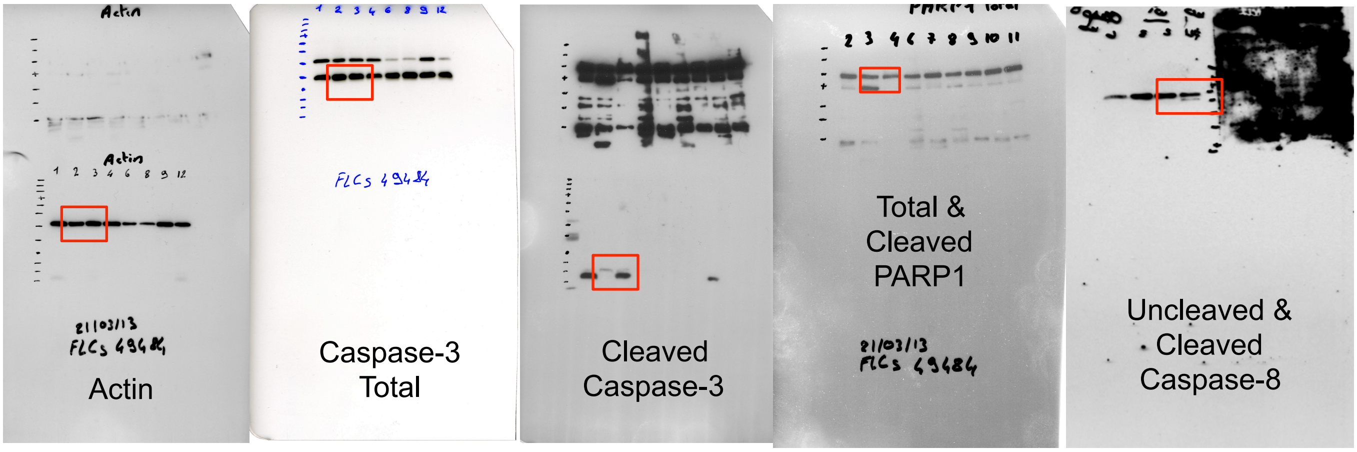

Figure S1b:

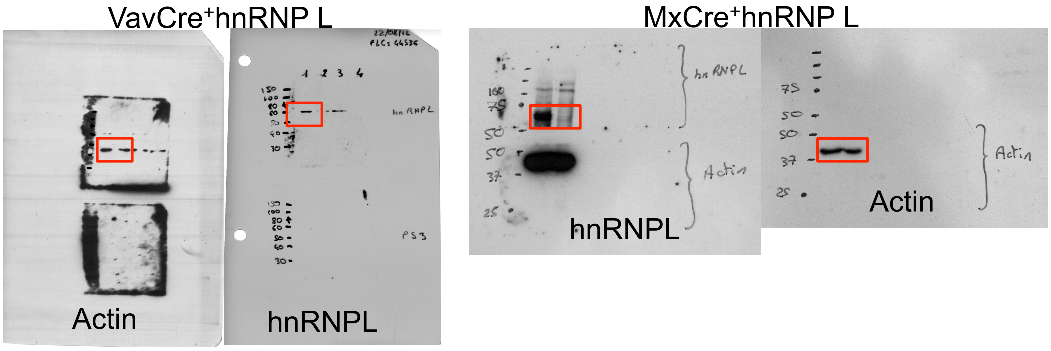

Figure S8a:

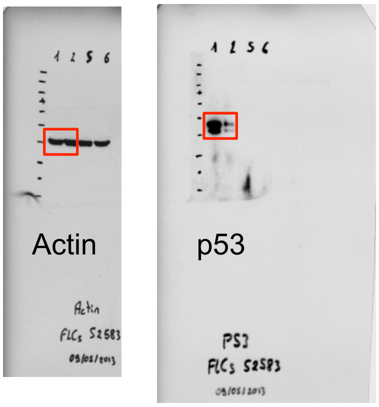

Supplemental Figure S11:
Uncropped images of the Western blots membranes used in Figures 7c, 7d, S1b, S8a. Red boxes highlight the cropped region of the membranes.

Supplemental Table S1:

Primers for the amplification of mouse genome regions from ChIP by real-time quantitative PCR

Name	Forward (5'-3')	Reverse (5'-3')
Trailr2_0	TGTTCAGTGTTCCAAACGTTACA G	ATGGATGGTCTCACCCCAGTA
Trailr2_1	TTCAGATTCTGTCCGGCGAC	GCTTGATTACGCGTCCAACC
Trailr2_2	CCTCCCAAGGTGGATTTCCC	GCGACTTGACGCTTAGTGGT
Trailr2_3	GTTTCGGATGAGCTGACACC	TAGAAGTCTGCGCTTCGGG
Trailr2_4	TCCGCGTCACATCACTGAC	AGGCCCGATTCTAGGCTCTT
Intergenic	TGGGCATATCCCTGGAGCTT	GGCCATCCCACAGTCACAAC

Primers for the amplification of mouse transcripts by real-time quantitative PCR

Name	Forward (5'-3')	Reverse (5'-3')
CD34	ACAGTACCTCACAACCCTGC	GGTCACATTGGCCTTTCCCT
cdkn1a/p21	TTGCACTCTGGTGTCTGAGC	AATCTGTCAGGCTGGTCTGC
Csf1r	GGTTGTAGAGCCGGGTGAAA	TCTTGTGGTCAGGGTGCTTC
Csf3r	CTGCTCCATCCCCCGAAAAA	TGAGAGACTACATCAGGGCCA
CXCR4	TTACCCCGATAGCCTGTGGA	CAGGAGAGGATGACGATGCC
Gfi1	GCGAGTGATTATTGGCCGC	TAGCTTTTGGGGGTTAGGGC
Meis1	ACCCTGATGGACAGCCAATG	CTGGCATACTTTGCAGCCCT
Notch1	CCTCCTGCCAGACCAAT	TCACTCTCACAGTTGCGACC
PU.1	ACTGGGATTTCTCCGCACAC	GCACCATGGGAGTATCGAGG
Runx1	AGGCAGGACGAATCACACTG	TGCTGTCTGAAGCCATCGTT
TrailR2	ACTGACTTCCTGTACCCCCA	GATCCCGTTCACAGCCTCTT
Trp53inp	CTGCCACAAGAAAGGGAGAG	ACAGATGCCACCAGAAATCC
P53	AAGACAGGCAGACTTTTCGCC	CGGGTGGCTCATAAGGTACC
GAPDH	ACTGAGCAAGAGAGGCCCTA	TATGGGGGTCTGGGATGGAA

Supplementary Table S2 - Genes with one or more exons showing significant differences in usage

Ensembl Gene ID	Gene Symbol	Gene name
ENSMUSG00000026817	ak1	adenylate kinase 1
ENSMUSG00000028737	ALDH4A1	aldehyde dehydrogenase 4 family, member A1
ENSMUSG00000040174	Alkbh3	alkB, alkylation repair homolog 3 (E. coli)
ENSMUSG00000026987	Baz2b	bromodomain adjacent to zinc finger domain, 2B
ENSMUSG00000032589	BSN	bassoon
ENSMUSG00000030177	Ccdc77	coiled-coil domain containing 77
ENSMUSG00000035776	CD99L2	CD99 antigen-like 2
ENSMUSG00000039128	Cdc123	cell division cycle 123 homolog (S. cerevisiae)
ENSMUSG00000032997	CHPF	chondroitin polymerizing factor
ENSMUSG00000026814	ENG	endoglin
ENSMUSG00000069808	FAM57A	family with sequence similarity 57, member A
ENSMUSG00000090639	Gm20425	predicted gene 20425
ENSMUSG00000094439	Gm21969	predicted gene 21969
ENSMUSG00000095538	Gm21983	Predicted gene 21983
ENSMUSG00000024958	GPR137	G protein-coupled receptor 137
ENSMUSG00000025001	Hells	helicase, lymphoid specific
ENSMUSG00000026618	iars2	isoleucine-tRNA synthetase 2, mitochondrial; similar to isoleucine-tRNA synthetase 2, mitochondrial
ENSMUSG00000041025	IFFO2	intermediate filament family orphan 2
ENSMUSG00000031171	LOC100044636	similar to Ftsj homolog; FtsJ homolog 1 (E. coli)
ENSMUSG00000089661	MIA	melanoma inhibitory activity
ENSMUSG00000093348	Mir3101	microRNA 3101
ENSMUSG00000032456	NMNAT3	nicotinamide nucleotide adenylyltransferase 3
ENSMUSG00000026211	Obsl1	obscurin-like 1
ENSMUSG00000019876	Pkib	protein kinase inhibitor beta, cAMP dependent, testis specific
ENSMUSG00000020612	PRKAR1A	protein kinase, cAMP dependent regulatory, type I, alpha
ENSMUSG00000061136	Prpf40a	PRP40 pre-mRNA processing factor 40 homolog A (yeast)
ENSMUSG00000026869	PSMD5	proteasome (prosome, macropain) 26S subunit, non-ATPase, 5
ENSMUSG00000072946	PTGR2	prostaglandin reductase 2
ENSMUSG00000000876	PXMP4	peroxisomal membrane protein 4
ENSMUSG00000053291	RAB4B	RAB4B, member RAS oncogene family
ENSMUSG00000028677	Rnf220	ring finger protein 220
ENSMUSG00000032553	srprb	signal recognition particle receptor, B subunit
ENSMUSG00000028738	Tas1r2	taste receptor, type 1, member 2
ENSMUSG00000056130	Ticam2	toll-like receptor adaptor molecule 2
ENSMUSG00000033184	TMED7	transmembrane emp24 protein transport domain containing 7
ENSMUSG00000032554	TRF	transferrin
ENSMUSG00000005354	TXN2	thioredoxin 2
ENSMUSG00000096115	NA	Uncharacterized protein

This table shows genes that have at least one exon with a greater than 2 fold idfference in usage in hnRNPL KO samples compare to hnRNPL wild-type samples, with a P value < 0.01 and at least 10 reads for quantification

Supplementary Table S3 - Most overrepresented Biological Functions of genes showing DEU in hnRNPL KO samples

Term
GO:0055114 oxidation reduction
GO:0043414 biopolymer methylation
GO:0022402 cell cycle process
GO:0032259 methylation

This table shows the GO Biological Functions most overrepresented amongst genes showing DEU in hnRPNL KO samples versus hnRNPL wild-type samples, according to the DAVID software

Note that none of these functions pass a 0.05 P Value threshold
